# Autoimmune pulmonary alveolar proteinosis in a patient with sarcoidosis

**DOI:** 10.1002/ccr3.2068

**Published:** 2019-03-02

**Authors:** Yuko Tanaka, Toshihiro Shirai, Kazuhiro Asada, Aya Muramatsu, Mineo Katsumata, Takafumi Suda

**Affiliations:** ^1^ Department of Respiratory Medicine Shizuoka General Hospital Shizuoka Japan; ^2^ Department of Pathology Shizuoka General Hospital Shizuoka Japan; ^3^ Second Division, Department of Internal Medicine Hamamatsu University School of Medicine Hamamatsu Japan

**Keywords:** autoimmune pulmonary alveolar proteinosis, crazy‐paving appearance, sarcoidosis

## Abstract

Steroids used to treat sarcoidosis may induce aPAP. The cooccurrence of sarcoidosis and autoimmune pulmonary alveolar proteinosis (aPAP) is rare. In this case, aPAP merged into sarcoidosis regardless of the disease state of sarcoidosis and improved naturally. When using steroids during sarcoidosis exacerbation, attention should be paid to aPAP relapse.

## 1 INTRODUCTION

The cooccurrence of sarcoidosis and autoimmune pulmonary alveolar proteinosis (aPAP) is rare. Steroids used to treat sarcoidosis may induce aPAP. Here, we report a rare case of aPAP complicating sarcoidosis. When using steroids during sarcoidosis exacerbation, attention should be paid to aPAP relapse.

Pulmonary alveolar proteinosis (PAP) is a disease in which eosinophilic granular proteinaceous substances abnormally accumulate in the alveolar space because surfactant production or degradation ability is impaired. Autoimmune PAP (aPAP) is the most common form of PAP. aPAP in patients with sarcoidosis has rarely been reported.^1^, ^2^, ^3^ Steroids used to treat sarcoidosis and immunosuppressants used to treat autoimmune disease may induce aPAP.^3^ However, the clinical course and histopathologic features of aPAP complicating sarcoidosis are not fully understood. Here, we report a case of aPAP that merged into sarcoidosis.

## 2 CASE PRESENTATION

**Figure 1:**
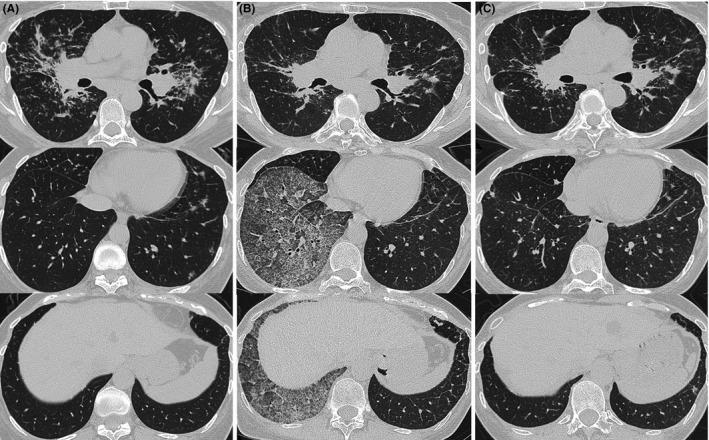
High‐resolution computed tomography images on the first visit showed bilateral multiple perilymphatic nodules (A). Eight years later, a crazy‐paving appearance was seen on the right lower lobe (B). Four months later, after video‐assisted thoracic surgery (VATS), the crazy‐paving appearance was markedly improved (C)

**Figure 2:**
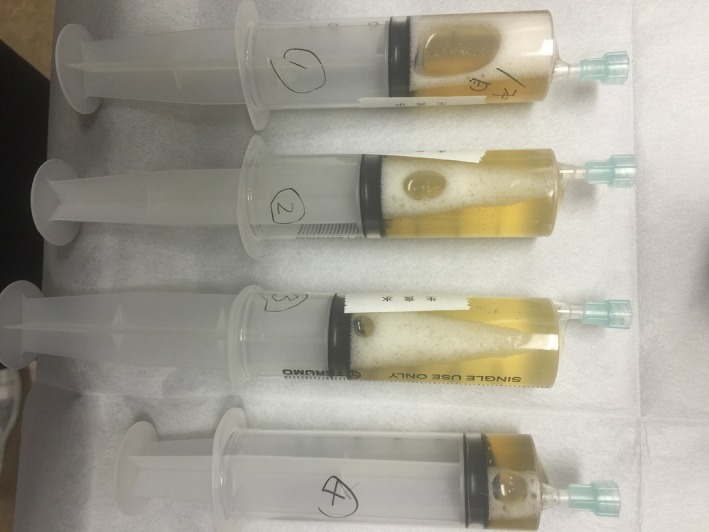
The bronchoalveolar lavage fluid (BALF) was yellow with a lymphocyte fraction of 96.8%

**Figure 3:**
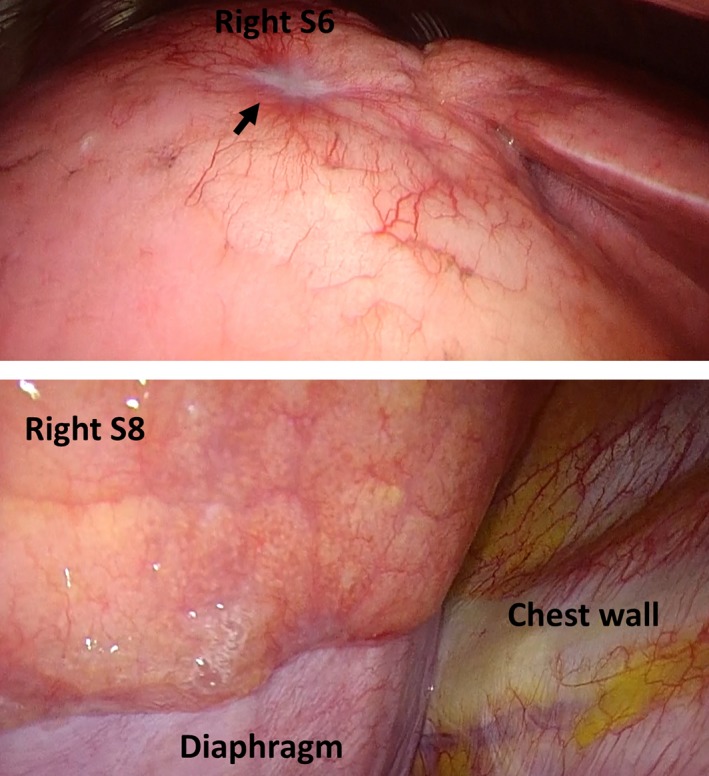
Video‐assisted thoracic surgery images. A white nodule that appeared to be a sarcoid nodule was found on the surface of the lung (arrow). The surface of the right lower lobe was the same yellow color as the BALF and was swollen due to the liquid stored in the alveoli

**Figure 4:**
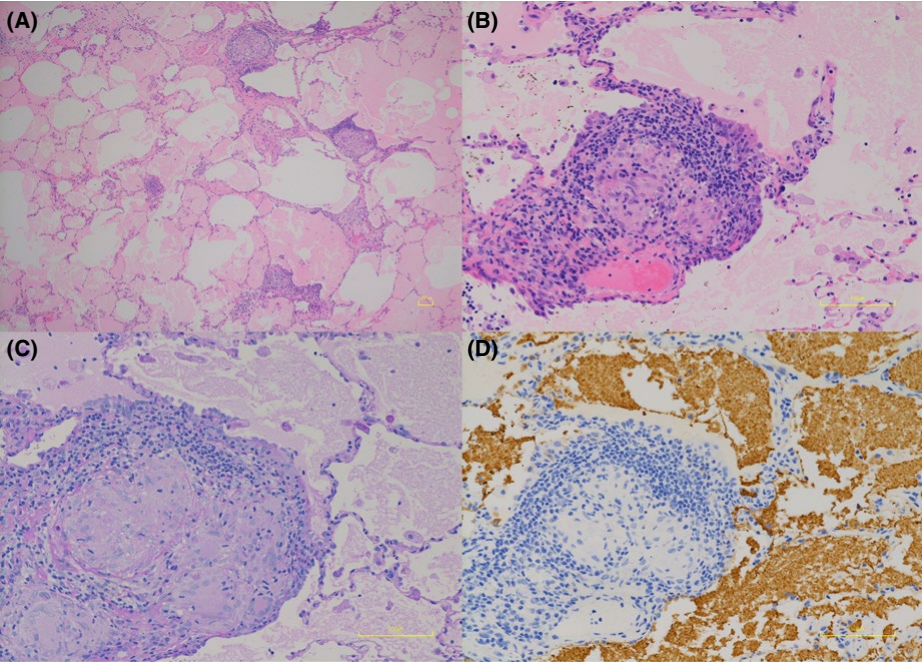
Histopathology showed that the alveolar cavity in contact with noncaseating granulomas along the lymphatic pathway was filled with a granular substance, and lipoid clefts were also seen in the right lung S8 (A, B). SP‐A staining was positive (C), and PAS staining was weakly positive (D). Foamy macrophages that seemed to phagocytize the granules were positive for both SP‐A and PAS staining (C, D)

A 58‐year‐old woman who was diagnosed with sarcoidosis eleven years prior was referred for the exacerbation of mediastinal lymph node enlargement, consolidation and multiple nodules on high‐resolution computed tomography (HRCT) eight years ago (Figure 1A). Pulmonary function was normal, and no subjective symptoms were noted; thus, the patient was followed up without medication. However, dyspnea developed gradually, and HRCT showed a crazy‐paving appearance in the right lower lobe (Figure 1B). Fine crackles were audible over the posterior right lung. Laboratory data revealed that KL‐6 levels increased to 630 U/mL and SP‐D levels to 255 ng/mL; however, the ACE level remained the same at 23.1 IU/L. Complications such as cardiac sarcoidosis occurred if sarcoidosis was exacerbated; thus, positron emission tomography (PET‐CT) was performed. However, no new lesions were found. The condition of pulmonary sarcoidosis did not become worse. Pulmonary function tests showed a restrictive ventilatory impairment (FVC 1.87 L, %FVC 62.3% predicted) and reduced diffusing capacity of the lung for carbon monoxide (DLCO 9.36 mL/min/mm Hg, %DLCO 55.5% predicted). The bronchoalveolar lavage fluid (BALF) was yellow with a lymphocyte fraction of 96.8% (Figure 2). Microscopic examination from transbronchial lung biopsies only found a tiny granuloma consistent with sarcoidosis. The crazy‐paving appearance did not improve after one month. A video‐assisted thoracoscopic lung biopsy was performed from right S6 and S8 to confirm the diagnosis. Observation under thoracoscopy revealed that the surface of the right lower lobe was yellow and swollen by the liquid stored in the alveoli (Figure 3). Histopathologic examinations revealed many perilymphatic noncaseating granulomas in both S6 and S8. In S8, the alveolar cavity was filled with a granular substance, periodic acid‐Schiff (PAS) staining was weakly positive, surfactant protein A (SP‐A) staining was positive and lipoid clefts were also seen. Foamy macrophages that seemed to phagocytize the granules were positive for both PAS staining and SP‐A staining (Figure 4). The sample was positive for anti‐GM‐CSF antibodies (4.8 μg/mL, cut‐off value is 1.0 μg/mL), and the patient was diagnosed with aPAP. Steroid administration was planned if the cause of the crazy‐paving appearance was the exacerbation of sarcoidosis, but we decided to follow her up because the diagnosis was aPAP. After four months, the crazy‐paving appearance went into spontaneous remission (Figure 1C). The laboratory data revealed that KL‐6 and SP‐D levels were 438 U/mL and 73.5 ng/mL, respectively, and pulmonary function tests showed the following improved values: FVC 2.03 L, %FVC 71.2% predicted, DLCO 11.46 mL/min/mm Hg and %DLCO 66.6% predicted.

## 3 DISCUSSION

We found that aPAP merges into sarcoidosis regardless of the disease state of sarcoidosis and may improve naturally. Some cases of aPAP with sarcoidosis have been reported.^1^, ^2^, ^3^ Mirchandani et al reported the first case report of combined PAP and sarcoidosis diagnosed in the same patient at first presentation. Whole lung lavage (WLL) under general anesthetic was performed. However, the patient did not require treatment for sarcoidosis and her spirometry remained stable.^1^ Boerner et al reported a 55‐year‐old woman who developed sarcoidosis after 15 months from WLL for the treatment of aPAP.^2^ Additionally, Yamasue et al reported a 64‐year‐old woman who developed aPAP after 13 months from treatment with steroids and immunosuppressive drugs for sarcoidosis and scleroderma who was successfully treated with the reduction of immunosuppressant therapy and repeated segmental BAL.^3^ From these reports, there was no relationship between sarcoidosis and the aPAP occurrence sequence. It became clear that aPAP merges into sarcoidosis regardless of the disease state of sarcoidosis. It has been reported that the value of anti‐GM‐CSF antibodies does not correlate with the disease state of PAP.^4^ However, in a previous report,^3^ showing bilateral extensive CT findings, the value of anti‐GM‐CSF antibodies was 11 309 μg/mL (measured with the same assay), which was much higher than in this case. Further studies with more similar cases will disclose the meaning of the value of anti‐GM‐CSF antibodies. The clinical course of aPAP varies and spontaneous improvement is observed in only 8% of patients.^5^ In these three cases, treatments were carried out for aPAP. However, in this case, the crazy‐paving appearance was localized, and the symptoms were mild; during follow‐up, the crazy‐paving appearance went into spontaneous remission after four months. When PAP recurs without respiratory failure in this case, we will follow without treatment. However, when respiratory failure occurs, whole lung lavage or segmental lung lavage will be considered. Furthermore, immunomodulating therapy (subcutaneous GM‐CSF, inhalation of GM‐CSF, Rituximab, or plasmapheresis) may also have to be considered in accordance with the previous reports.^2^, ^3^

Additionally, aPAP with sarcoidosis presents a crazy‐paving appearance in HRCT images. Johkoh et al reported three types of processes that resulted in crazy‐paving appearance, including an alveolar filling process, interstitial fibrotic process, and a combination of both.^6^ In this case, noncaseating granulomas in a perilymphatic distribution and mild fibrosis of the alveolar septa were observed in the right lung S6 and S8. However, intra‐alveolar fluid was observed only in the right lung S8. Furthermore, the crazy‐paving appearance in HRCT images was seen only in the right lung S8. Therefore, the crazy‐paving appearance in this case was thought to be due to an alveolar filling process. In this case, the crazy‐paving appearance in aPAP was confined to the right lower lobe, and it was independent of perilymphatic distribution of nodules in sarcoidosis. Chronic immune responses such as activated macrophages and cytokines are involved in the formation of sarcoidosis granuloma,^7^ while aPAP is an autoimmune disease by anti‐GM‐CSF antibodies. Different independent mechanisms could have occurred simultaneously in case of aPAP with sarcoidosis.

## 4 CONCLUSION

Autoimmune pulmonary alveolar proteinosis (aPAP) merges into sarcoidosis regardless of the disease state of sarcoidosis and may improve naturally. A crazy‐paving appearance can be found for various reasons. In aPAP associated with sarcoidosis with multiple perilymphatic nodules, the crazy‐paving appearance is due to an alveolar filling process. When using steroids during sarcoidosis exacerbation, attention should be paid to aPAP relapse.

## CONFLICT OF INTEREST

I declare, on behalf of my coauthors and myself, that we do not have any conflicts of interest. Appropriate written informed consent was obtained for the publication of this case report and accompanying images.

## AUTHOR CONTRIBUTION

YT: took care the patient and wrote the manuscript as the primary author. TS: took care and followed up the patient and critically reviewed and supervised the manuscript. KA: participated in patient care. AM and MK: provided pathological diagnosis of the patient. TS: supervised the manuscript.
